# Canine Cardiac and Cardiovascular Pathology: Four Major Life-Threatening Non-Degenerative, Non-Hereditary Conditions

**DOI:** 10.3390/vetsci12111060

**Published:** 2025-11-04

**Authors:** Adrian Stancu, Radu-Valentin Gros, Iasmina Luca, George-Andrei Călugărița, Alexandru Gavrilă, Aurelian-Sorin Pașca

**Affiliations:** 1 Faculty of Veterinary Medicine, Department of Anatomic Pathology and Forensic Medicine, University of Life Sciences “King Mihai I” from Timisoara, Calea Aradului 119, 300645 Timișoara, Romania; adrianstancu@usvt.ro (A.S.); george-andrei.calugarita.fmv@usvt.ro (G.-A.C.); gavrilaalexandruioan@gmail.com (A.G.); 2 Faculty of Veterinary Medicine, Department of Microbiology, University of Life Sciences “King Mihai I” from Timisoara, Calea Aradului 119, 300645 Timișoara, Romania; 3 Faculty of Veterinary Medicine, Department of Anatomic Pathology and Forensic Medicine, Iasi University of Life Sciences, Aleea Mihail Sadoveanu nr. 3, 700490 Iaşi, Romania; sorin.pasca@iuls.ro

**Keywords:** dog, cardiovascular diseases, parvovirus, *Dirofilaria*, hemangiosarcoma, polyarteritis

## Abstract

**Simple Summary:**

Cardiovascular diseases are particularly significant in dogs, often causing life-threatening lesions. However, some conditions, such as polyarteritis nodosa, remain poorly understood. In this review, we present several conditions of varied etiology, described over time in the specialized literature, and interesting lesions, with a strong visual impact, from our forensic medicine clinic.

**Abstract:**

Cardiovascular diseases in dogs have diverse causes and may progress rapidly to life-threatening complications. This review outlines the relevant pathological conditions involving the cardiovascular system in dogs, especially the myocardium, including myocarditis caused by canine parvovirus (CPV-2), heartworm disease (*Dirofilaria immitis*), hemangiosarcoma, and polyarteritis nodosa (PAN). CPV-2 affects the myocardium of puppies during the early weeks of life, leading to necrosis, fibrosis, and congestive heart failure. Heartworm disease is caused by adult *D. immitis* residing mainly in the pulmonary arteries, inducing pulmonary hypertension, right ventricular overload, and vascular damage, with the severity being related to the worm burden and duration of infestation. Hemangiosarcoma is a malignant vascular tumor, most frequently originating in the spleen or right atrium, often diagnosed at an advanced stage, with widespread metastases. Polyarteritis nodosa in dogs is a necrotizing, systemic vasculitis of medium-sized arteries that may affect the coronary arteries of the heart. Its pathogenesis is still unclear, though an immune-mediated mechanism is suspected. By presenting these lesions, the review underscores the many factors that can trigger cardiovascular diseases in dogs, as well as the clinical significance and the need for further research into their pathogenesis and treatment.

## 1. Introduction

Canine parvovirus (CPV) is a virus frequently isolated from dogs, known for its infectivity, pathogenicity, and high mortality rate in the canine population. CPV exists as two serotypes, CPV-1 and CPV-2, and is a small (18–26 nm), icosahedral, non-enveloped virus with a linear, single-stranded DNA genome. CPV-2 primarily infects rapidly dividing cells in the bone marrow, small intestines and heart muscle, which can lead to canine parvovirus enteritis (PVE) and canine myocarditis. Perinatal infections are responsible for extensive myocardial necrosis in affected puppies, which can result in either acute, high mortality or progressive cardiac injury. However, many veterinarians believe that the extensive use of vaccines has significantly reduced the incidence of canine parvoviral myocarditis in dogs [1,2,3,4,5,6,7].

*Dirofilaria immitis* is a common nematode worldwide, which frequently causes cardiovascular infestations with a high mortality rate in canids. Heartworms are frequently encountered in the pulmonary artery and right ventricle. However, since 1856, adult heartworm infestations have been reported in abnormal sites such as the eye, peritoneal cavity, subcutaneous region, skeletal muscle, systemic arteries and the central nervous system [3]. The most common lesions are right ventricular dilatation or cardiac hypertrophy associated with lung inflammation, cough, dyspnea, exercise intolerance and fatigue. Ascites and congestive chronic hepatomegaly may also be seen in patients with right heart failure [2,8,9].

Polyarteritis nodosa (PAN), also referred to as panarteritis, periarteritis nodosa, juvenile polyarteritis, or Beagle Pain Syndrome (BPS), is a non-infectious disease affecting primarily medium-sized muscular arteries as well as small peripheral and visceral vessels. It is characterized by segmental and disseminated lesions distributed along branching vessels, most often at points of arterial bifurcation. It is a complex pathological process that, in a chronic progression, leads to vascular wall damage. The pathogenesis of PAN remains poorly understood, although an immune-mediated mechanism is suspected, placing it among diseases with an (auto)immune pathogenesis [10,11,12,13].

Hemangiosarcoma (HSA) is a malignant endothelial neoplasia that is suspected to originate from a pluripotent bone marrow progenitor with a complex and multifactorial pathogenesis [8]. HSA is a highly aggressive cancer with a high prevalence in dogs. While cutaneous tumors can often be effectively managed by surgical excision, visceral forms are usually incurable. Therapeutic advances have been hampered by limited understanding of the biology underlying the disease. The spleen, right atrium of the heart, subcutaneous tissue, and dermis are the most common primary sites for HSA [14,15,16].

A comparative analysis of the main cardiac conditions discussed in this article, based on cases diagnosed at the necropsy clinic of our faculty over a four-year period, is presented in Table 1. The conditions included are parvoviral myocarditis, heartworm disease, cardiac hemangiosarcoma and polyarteritis nodosa. Parvoviral myocarditis was observed primarily in very young puppies, between 8 and 12 weeks of age, without evidence of breed predisposition, and was also diagnosed in mixed-breed dogs. Heartworm disease was diagnosed predominantly in adult dogs, ranging in age from 6 to 14 years, with a higher incidence in outdoor dogs. Cardiac hemangiosarcoma was most frequently identified in senior dogs (7–15 years) and showed a clear predisposition for large breeds, particularly Labrador Retrievers, German Shepherds, Golden Retrievers, and Boxers. In the case of hemangiosarcoma, the highest incidence is in the heart (right atrium) and spleen. In approximately 25% of cases, both organs are affected simultaneously. However, metastases can occur in the lung, small intestine and rarely in the brain. Polyarteritis nodosa was mainly observed in young dogs, aged 4 to 24 months, with Beagles being the most commonly affected breed, followed by Dachshunds and Boxers.

**Figure 1:**
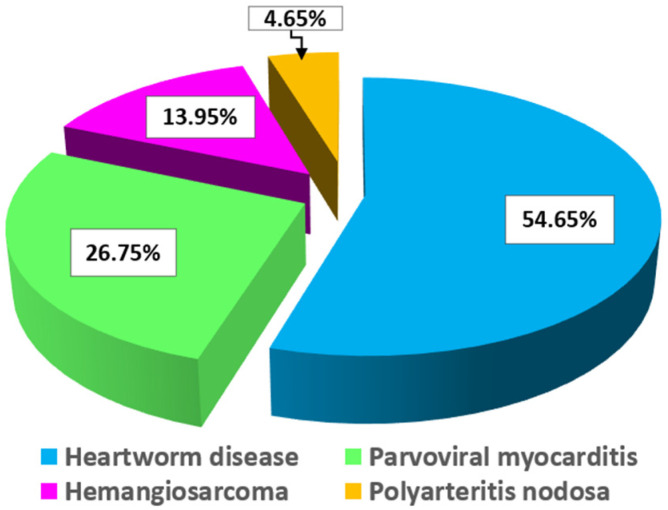
Incidence of Cardiac Diseases in Our Necropsy Clinic Over a 4-Year Period.

Overall, the data presented in the table highlight the significant role of age and breed predisposition in the occurrence of heart disease in dogs. The incidence of these cardiac conditions in dogs is also presented graphically in Figure 1.

The characteristic histopathological features of each condition, the animals’ clinical signs, and the challenges encountered in establishing the diagnosis are summarized in Table 2. We note that all histopathological elements presented in Table 2 have been observed in our practice; however, they are not necessarily present in their entirety in every histopathological specimen derived from the same cardiac conditions in dogs.

## 2. Parvoviral Myocarditis

### 2.1. Etiopathogenesis

Canine parvovirus (CPV) belongs to the family *Parvoviridae*, genus *Protoparvovirus*. Among the serotypes and variants of CPV are CPV-1, discovered in the early 1960s, and CPV-2, discovered in the 1980s, which underwent numerous antigenic changes. Today, CPV variants (2a, 2b, 2c) circulate concomitantly in canine populations [17]. CPV-1 can cross the placental barrier in pregnant bitches, and depending on the period in which the female contracted the virus, it may be responsible for spontaneous abortions or may be transmitted to the fetus, so that puppies may develop myocarditis in the first 12 weeks postpartum [7,17,18,19].

The age of dogs testing positive for CPV-2 indicates a broad window of susceptibility to cardiac complications caused by CPV-2. Parvoviruses depend on the cellular processes of actively dividing cells in the S-phase for replication. Thus, the heart’s susceptibility to CPV infection may be linked to the high rate of DNA synthesis in cardiomyocytes of newborn animals, followed by a decrease as they mature. Viral VP2 mRNA was maximal in puppies, particularly those aged between three weeks and two months. However, significant viral mRNA was also detected in the hearts of older puppies, with the ISH signal being identified in cardiomyocytes of dogs aged up to 84 days [1,18,19,20].

Research on the development of the cardiac muscle in dogs has indicated that factors promoting CPV-2 replication are present from birth until weaning. At the same time, there are significant variations in growth patterns between breeds, depending on breed type and other factors, which may influence heart development and the likelihood of developing CPV-2 myocarditis [20,21].

Other researchers suggest that the severity of myocarditis in puppies infected with canine parvovirus may vary depending on viral strain, immune response, and concurrent infections. Some studies indicate that early medical intervention and supportive treatment can improve survival rates, while others emphasize the need for further research to fully understand the long-term cardiac effects of the infection [4,19,20,22,23,24].

### 2.2. Clinical Signs

Cardiac degenerations mainly lead to congestive heart failure associated with pulmonary edema and chronic hypoxia [16]. However, the brain may undergo irreversible degenerative processes such as leukoencephalomalacia and necrotizing vasculitis [1,25]. Ascites and hydrothorax were also detected in most puppies [3,20,23].

### 2.3. Gross Lesions

Studies have shown that affected individuals may develop cardiac hypertrophy, with the heart representing 1.33% of total body weight compared to 0.7–0.8% as predicted in adult and newborn dogs. Research conducted at Wisconsin Veterinary Diagnostic Laboratory revealed dilated cardiomyopathy with multifocal and locally extensive areas of myocardial pallor in both ventricles. Discoloration of the ventricular walls was also observed, along with decreased elasticity. The left ventricular free wall was thin, measuring 5 mm, while the right ventricular free wall was even thinner, measuring 2.5 mm. Approximately 5 mL of sero-haematic collection was present in both the thoracic and abdominal cavities [20,22].

### 2.4. Histological Features

Multifocal myocardial necrosis and degeneration with basophilic intranuclear inclusion bodies in cardiomyocytes and lymphocytic to granulomatous myocarditis, as well as progressive myocardial fibrosis, in chronic cases, were observed in most affected puppies [20,22]. In some individuals, a mixed inflammatory process consisting of neutrophils, lymphocytes, and plasma cells was reported, while in others a localized suppurative process was present [1]. Stancu et al. identified viral inclusions in cardiomyocytes associated with enteritis caused by CPV-2 (study currently under peer review). Typically, the simultaneous occurrence of intestinal and cardiac disease is not commonly observed in spontaneously infected animals or laboratory experiments. However, it has been experimentally demonstrated that viruses obtained from myocardial tissues can induce enteritis [1,20,22].

### 2.5. Diagnostic Methods

Identification of viral inclusions in cardiomyocytes supports the diagnosis of CPV myocarditis. Detection of viral VP2 mRNA by in situ hybridization (ISH) demonstrated the presence of CPV in the cardiomyocytes of puppies up to 84 days old [20,21,26]. These findings underline the potential of molecular diagnostic methods in establishing the role of CPV in myocardial disease [4,6,17,21,26].

## 3. Heartworm Disease

### 3.1. Etiopathogenesis

*Dirofilaria* spp. infections in dogs are widespread mosquito-borne parasitic infections caused by filarioid nematodes (*Onchocercidae*), namely *Dirofilaria immitis* and *Dirofilaria repens*, both of which have zoonotic potential [27,28,29].

*Dirofilaria immitis* and *Dirofilaria repens* are two well-known vector-borne helminths that pose significant challenges to veterinary medicine and public health due to the continuous increase in the number of cases among the canine population [2,28,30,31].

Microfilariae of *Dirofilaria* spp. are released by adult females into the host’s bloodstream and are ingested by a competent mosquito during blood feeding [28]. Within the mosquito, the microfilariae undergo sequential development from L1 to L2 and subsequently to the infective L3 stage. The infective L3 larvae migrate to the mosquito’s proboscis and salivary glands and are transmitted to a new vertebrate host during a subsequent blood meal. In the vertebrate host, the larvae molt to the L4 stage and ultimately develop into immature and adult parasites. Multiple mosquito genera, including *Aedes* spp., *Culex* spp., and *Anopheles* spp., have been experimentally or field-verified as competent vectors, permitting the development of *Dirofilaria immitis* to the infective L3 stage. Vector competence and the rate of larval development are strongly influenced by temperature and mosquito species [32].

Humans are considered accidental hosts for *Dirofilaria immitis*. Infection is generally limited and of low pathogenicity, typically presenting as solitary subcutaneous nodules or pulmonary “coin” lesions, which are often asymptomatic or accompanied by mild respiratory symptoms such as chest pain, cough, or low-grade fever. These lesions can mimic neoplastic processes on imaging studies. Nevertheless, due to the potential for radiologic misinterpretation as tumors, histopathologic or surgical confirmation is frequently required [33].

The pathogenesis of heartworm disease is influenced by the worm burden, the host’s immune response, duration of infection, and the level of physical activity [34].

The primary lesion occurs in the pulmonary arteries and lung parenchyma and is usually generated by the adult stage of parasites. If untreated, they can cause pulmonary hypertension, leading to extra pressure on the right side of the heart, which can cause right ventricular dysfunction, arrhythmias, and ultimately, right-sided heart failure [32,35,36].

### 3.2. Clinical Signs

Heart disease has many clinical manifestations and is caused by both adult parasites and their larval stages (microfilariae). Adult heartworms primarily inhabit the pulmonary arteries, where they induce the most significant pathological changes, and in cases of heavy worm burdens, may extend into the right ventricle [30].

Other symptoms may be correlated with the blockage of blood flow due to the presence of *D. immitis* on the tricuspid valve. In rare cases, aberrant migration of adult worms occurs, resulting in their presence in an atypical location such as the epidural space, brain, anterior chamber of the eye, lung parenchyma, or systemic arteries. These unusual locations are frequently associated with severe or advanced infestation and can result in localized inflammation, obstruction, or other organ-specific complications. There have been cases where *D. immitis* was found in association with a nerve sheath tumor heartin a dog [31,37].

### 3.3. Gross Lesions

In chronic evolutions, vasculitis and vascular sclerosis were also noted in multiple organs [34]. Even necrotizing vasculitis in the femoral artery and abdominal aorta may appear in case of erratic migrations [9,37].

Some authors mentioned in their studies minimal damage to the heart, with a reduced inflammatory reaction in the pericardium, resulting from the presence of calcified adult parasitic forms [35].

Chronic *Dirofilaria immitis* infection in dogs can lead to immune complex–mediated glomerulopathies. Clinical and experimental studies have demonstrated deposition of immune complexes and electron-dense deposits in the glomeruli, exhibiting membranous and membranoproliferative patterns. These lesions result from circulating dirofilarial antigens complexing with host antibodies, leading to deposition in the glomerular basement membrane, which contributes to proteinuria and impaired renal function. Additionally, infection may elicit pulmonary granulomatous reactions characterized by eosinophil-rich inflammation and granuloma formation. Eosinophilic pulmonary granulomatosis (EPG) represents a clinicopathologic syndrome that can be associated with, but is not always caused by, heartworm infection [12,38].

### 3.4. Histological Features

Most researchers have reported advanced degeneration of the vascular wall in the lung parenchyma, with a lympho-histiocytic infiltrate at the endothelial level with or without pseudopapillary hyperplasia [35]. In certain cases, advanced pneumonia with multifocal interstitial nodular infiltrate in the pulmonary parenchyma was noted. However, no peribronchiolar infiltrate was observed, as cited by some authors. This finding supports the hypothesis that the severity of vascular injury is related to the parasite load and time of parasitism [35,39,40].

### 3.5. Diagnostic Methods

Combination therapy with doxycycline and a filaricide prior to melarsomine administration has been shown to reduce pulmonary vascular injury compared to treatment with doxycycline and melarsomine or melarsomine alone. Certain biomarkers may serve as reliable indicators for the follow-up and monitoring of pulmonary thromboembolism in canids infected with *Dirofilaria immitis*. These markers reflect inflammation, endothelial damage, and pulmonary hypertension, which are essential for the early detection of disease-induced damage [35,39,40].

## 4. Hemangiosarcoma

### 4.1. Etiopathogenesis

Hemangiosarcoma is a malignant tumor derived from vascular endothelial cells and is known to occur more frequently in senior dogs, particularly German Shepherds and Golden Retrievers [41,42]. Recent studies suggest that hemangiosarcomas may originate from a pluripotent bone marrow progenitor, highlighting their complex pathogenesis. Hemangiosarcoma can occur in any vascularized tissue but most commonly arises from the spleen, skin/subcutaneous tissue, liver, and right atrium in dogs, causing multiple organ metastases in both domestic and wild carnivores [43].

### 4.2. Clinical Signs

Cardiac hemangiosarcomas are primarily located in the right atrium, though they are occasionally found in the right ventricle or at the base of the heart. Clinically, right atrial hemangiosarcoma may present with signs of cardiac tamponade, including weakness, collapse, pale mucous membranes, and exercise intolerance. In certain cases, hemangiosarcoma may clinically present neurological signs due to brain metastases with possible secondary intracranial hemorrhages [42,44,45].

### 4.3. Gross Lesions

Cases of hemangiosarcoma involving the left coronary sulcus and periaortic region have been reported in African wild dogs [39,44,46,47]. When the spleen is involved, dogs can develop severe anemia and hemoperitoneum [48]. Yan et al. (2024) identified an interventricular septal hemangiosarcoma after necropsy, with myocardial necrosis and inflammation noticed in the left ventricle adjacent to the paraconal groove. Concurrent pulmonary and hepatic metastases were also present [42].

### 4.4. Histological Features

Histologically, three morphological subtypes are recognized: conventional/well-differentiated, kaposiform/spindle cell, and epithelioid [15].

Cardiac hemangiosarcoma is characterized by infiltration of myocardial fibers by neoplastic endothelial cells, resulting in myocyte atrophy and degeneration. Tumor cells exhibit pleomorphic, hyperchromatic nuclei with frequent mitoses and form irregular capillary-like vascular channels with associated hemorrhage. Similar neoplastic proliferation is observed in the spleen, liver, and lymph nodes, consistent with metastatic capillary hemangiosarcoma [49,50].

### 4.5. Diagnostic Methods

Diagnosis is based on clinical signs, imaging for cardiac masses, necropsy findings, and histopathological examination to identify subtype and confirm metastases [42].

## 5. Polyarteritis Nodosa (PAN)

### 5.1. Etiological Hypotheses

The exact etiology of polyarteritis nodosa (PAN) in dogs remains largely unknown. It is generally considered a systemic necrotizing vasculitis of suspected immune-mediated origin. Although the role of immune complexes in its pathogenesis is debated, some authors suggest that deposition of antigen–antibody complexes in the vascular walls may contribute to the development of vascular inflammation and damage. Infectious agents, chronic inflammatory conditions, and immune dysregulation have also been proposed as potential triggers, although no definitive causative agent has been consistently identified [13,51,52,53].

### 5.2. Predisposed Breeds

PAN has been reported sporadically in dogs, with several breeds, notably the Beagle, Boxer, Bernese Mountain Dog, Dachshund, and Welsh Springer Spaniel, considered predisposed, possibly due to underlying genetic factors. In Beagles, a juvenile form of polyarteritis syndrome is particularly recognized, most often in young individuals, and clinical manifestations generally show a favorable response to corticosteroid therapy [54,55].

### 5.3. Clinical Signs

Vascular changes on the surface of the heart, including thickening and dilation, have been reported at necropsy in a young dog [13]. Clinically, affected dogs may present with nonspecific signs such as fever, lethargy, and neurological manifestations depending on vascular involvement. An acute febrile episode was described in an elderly Corgi following removal of a subcutaneous arteriovenous fistula. The dog deteriorated into a coma after a week of illness and was euthanized [51,56].

### 5.4. Gross and Microscopic Lesions

The initial lesions in PAN consist of endothelial swelling. Plasma fibrin precipitation and fibrinoid degeneration within the tunica media typically progress to fibrinoid necrosis. The chemotactic properties of fibrin and fibrinoid promote massive infiltration of granulocytes (neutrophils, eosinophils) both within and around the affected vessel. Subsequently, fibrin is phagocytosed, granulocytes undergo degeneration, and fragmented cellular debris leads to acute leukocytoclastic vasculitis. The proteolytic enzymes released within the arterial wall may result in sac-like (saciform) aneurysmal dilations [13,56].

A case of cutaneous necrotizing vasculitis consistent with cutaneous PAN was described in a three-year-old dog presenting with a large dorsal subcutaneous mass that had been evolving for four weeks. Histologically, there was a discontinuous focal necrotizing vasculitis (fibrinoid type) associated with an intramural mixed inflammatory infiltrate comprising neutrophils, eosinophils, and mononuclear cells. Leukocytoclasis and thrombosis were also reported [51,54,57].

Carpenter et al. described an acute febrile episode in an elderly Corgi following the removal of a subcutaneous arteriovenous fistula. The dog deteriorated into a coma after a week of illness and was euthanized. Necropsy revealed lesions resembling PAN and those typically seen in human rheumatic heart disease. The vasculitis involved the coronary arteries and several small arteries in the liver, kidneys, spleen, gastrointestinal tract, urinary bladder, iris, choroid, meninges and brain. The lesions were segmental, with prominent fibrinoid necrosis and frequent thrombosis [51,56].

### 5.5. Diagnosis

Diagnosis of PAN is based on a combination of clinical findings, gross vascular changes, and histopathology revealing fibrinoid necrosis, leukocytoclastic vasculitis, and segmental arterial involvement. Advanced imaging and exclusion of infectious causes may support the diagnosis [13,51,53,54,56,57].

## 6. Conclusions

This review highlights the multifactorial nature of canine cardiac lesions, illustrating how viral, parasitic, neoplastic, and immune-mediated etiologies can converge to produce severe and often fatal conditions. By presenting these conditions, the article emphasizes the need for integrated diagnostic approaches, awareness of overlapping clinical signs, and further research into their pathogenesis. Such a comparative perspective supports veterinary medicine in recognizing, managing, and preventing the diverse causes of canine cardiac pathology.

Canine parvovirus (CPV-2) infection is a well-recognized cause of myocarditis in young dogs, often resulting in significant myocardial damage in the first weeks of life [4,20,58,59].

Canine heartworm disease remains a major health problem in Europe, with a significant zoonotic risk, even though its pathogenicity in humans is low [33,36,60].

Therefore, it is crucial to raise awareness among both veterinarians and physicians about the ongoing spread of these zoonotic filarial parasites [39].

Polyarteritis nodosa (PAN) causes multifocal fibrinoid necrosis, transmural inflammation, and subsequent fibrosis, affecting the entire vascular wall and surrounding tissues. Although its exact cause remains unclear, it is generally believed to result from immune complex deposition in small and medium-sized arteries, triggering a widespread inflammatory response [13,23,52,61].

Hemangiosarcoma is a malignant tumor that commonly arises from the right atrium, particularly in certain breeds of dogs, such as German Shepherds. This often-fatal neoplasm may initially present with nonspecific signs and symptoms similar to those of other organ systems (e.g., digestive or neurological disorders). In more advanced stages, it can also cause massive hemorrhagic effusion into the pericardial sac, leading to anemia, cardiac tamponade, and myocardial degeneration [45,47,50].

## Figures and Tables

**Table 1:** Prevalence of Major Cardiovascular Diseases in Dogs.

Disease	Number of Cases	Age	Breed Predisposition
Parvoviral myocarditis	23	8–12 weeks	No breed predisposition, also affects mixed breeds.
Heartworm disease	47	6–14 years	No breed predisposition, also affects mixed breeds.
Hemangiosarcoma	12	7–15 years	Boxer, German Shepherd, Golden Retriever, Labrador Retriever.
Polyarteritis nodosa	4	4–24 months	Beagle, Boxer, Dachshund.

**Table 2:** Comparative clinicopathological profile of cardiac diseases in dogs: prevalence, clinical signs, histological aspects and diagnostic challenges (4-year review).

Disease	Etiology	Prevalence	Clinical Signs	Key Histological Findings	Challenges
Parvoviral myocarditis	Canine parvovirus (CPV)	26.7%	Fatigue, dyspnea, cough, rarely ascites and syncope	Basophilic intranuclear inclusion bodies in cardiomyocytes.	The focal nature of the lesion means inclusion bodies may be transient, sometimes not being observed in the late stages of the disease; inflammatory processes and fibrosis may mask the lesional appearance.
Heartworm disease	*Dirofilaria* spp.	54.7%	Chronic cough and dyspnea, severe fatigue, tachycardia	The histopathological appearance is not characteristic; vascular wall degeneration and the presence of lymphohistiocytic infiltrate are dependent on the number of parasites.	Cardiac lesions may be secondary to right heart failure, not directly due to the parasite; requires correlation with clinical and necropsy context.
Hemangiosarcoma	Multifactorial (genetic, biological and environmental factors)	14.0%	Asymptomatic, sudden death	Blood vessels show irregular (abnormal) shapes, with vascular areas engorged with blood.	However, in some cases, the tumoral formations exhibit tight vascular anastomoses, lacking the typical vascular pattern characteristic of hemangiosarcoma, and displaying undifferentiated tumor cells. The differential diagnosis from other undifferentiated cell tumors is established by immunohistochemistry (IHC)
Polyarteritis nodosa (PAN)	Immune-mediated	4.6%	Febrile episodes, lethargy, neurological manifestations	It is not characteristic. Vasculitis with intramural and perivascular granulocytic infiltration.	The differential diagnosis must consider vasculopathies and blood vessel thrombosis. Identification of infectious agents is mandatory in the diagnosis of infectious vasculitis.

## Data Availability

No new data were created or analyzed in this study. Data sharing is not applicable to this article.
